# BUILDING BRIDGES AND CATALYZING RESEARCH FOR EVIDENCE-BASED INFECTION CONTROL: PROJECT ECHO FOR NURSING HOMES

**DOI:** 10.1093/geroni/igad104.2020

**Published:** 2023-12-21

**Authors:** Liza Behrens, Emily Heilbrunn, Marie Boltz

## Abstract

Nursing homes were ill-equipped for the pandemic; though facilities are required to have infection control staff, only 3% have taken a basic infection control course. Significant research has focused on infection control in the acute care setting; however, little is known about the implementation of infection control practices in long-term care communities. In this symposium, we will describe findings from the Project ECHO for Nursing Homes- a national, Patient-Centered Outcomes Research Institute (PCORI) funded study. First, Dr. Kraschnewski will present over all study findings from the stratified, cluster randomized controlled trial of 136 nursing homes that participated in up to 33 weeks of best-practice training to improve COVID-19 infection control practices. Next, Dr. Calo will present on implementation outcomes at 6-months including readiness for change and the implementation climate in the NH. Next, Ms. Heilbrunn will discuss the impacts of implementing evidence-based infection control practices through COVID-19 on NH residents’ well-being. Finally, Dr. Behrens will describe NH leadership perspectives (n=14) on preparing and maintaining care quality during times of diminishing resources. Data presented in this symposium can guide NHs in strategies to implement evidence-based infection control in their homes and informs researchers who are looking to implement similar implementation studies in the NH setting.

